# Large-group virtual anatomy via a smart digital podium: a preliminary cross-sectional study of student perspectives toward remote anatomage teaching in a medical lecture hall

**DOI:** 10.1186/s12909-026-10019-x

**Published:** 2026-07-25

**Authors:** Rohini Punja, Dhiren Punja, Ruben Prasad Kamachi Christdoss

**Affiliations:** 1 Department of Anatomy, Kasturba Medical College, Manipal Academy of Higher Education, Manipal, India; 2 Department of Physiology, Kasturba Medical College, Manipal Academy of Higher Education, Manipal, India

**Keywords:** Anatomy education, Virtual dissection, E-learning, Medical education, Technology-enhanced learning, Neuroanatomy, Basal nuclei, Blended learning, Competency-based medical education

## Abstract

**Background:**

The Anatomage virtual dissection table is conventionally deployed in small groups in the dissection laboratory, limiting simultaneous access to one cohort at a time. Remote streaming of the live Anatomage interface over an institutional local area network (LAN) to a smart digital podium in a large lecture theatre—termed Remote Anatomage Teaching (RAT)—represents an unexplored model for scaling virtual anatomy instruction to an entire medical cohort simultaneously.

**Methods:**

A cross-sectional study was conducted at Kasturba Medical College, Manipal, India. A single integrated basal nuclei lecture combining blackboard diagrams, PowerPoint slides, and live remote Anatomage streaming was delivered to 125 first-year MBBS students. A 13-item attitude questionnaire, developed de novo for this study, was completed anonymously by 94 students immediately post-session (response rate: 75.2%, 94/125); internal consistency of nine attitudinal preference items (the seven comparative items [Items 3, 4, 6–10], Interest in Anatomy [Item 11], and Curriculum Integration [Item 12], excluding Realism [Item 5]), used as a descriptive baseline reliability estimate, was Cronbach’s α = 0.873.

**Results:**

The RAT session was delivered without technical interruption throughout the 47-minute lecture. Among 94 respondents, 76.6% (72/94) rated RAT as more engaging or much more engaging than regular theory classes. RAT was preferred over PowerPoint-based lectures across all nine comparative pedagogical domains, with preferences ranging from 55.3% (self-paced learning) to 84.0% (curriculum integration). Realism compared to cadaveric dissection was endorsed by 81.9% (77/94). Three qualitative themes emerged from reflexive thematic analysis of 30 substantive free-text responses: visual engagement and retention, preference for blended delivery, and projection visibility as a technical constraint.

**Conclusions:**

Large-group RAT via a smart digital podium is technically feasible and elicits strongly favorable student perspectives across multiple pedagogical domains. This scalable model extends virtual anatomy technology to large lecture hall settings without additional hardware investment. A powered randomized controlled trial incorporating an active comparator arm is warranted as the definitive next step.

**Supplementary Information:**

The online version contains supplementary material available at https://doi.org/10.1186/s12909-026-10019-x.

## Background

Gross anatomy remains the structural and conceptual foundation of medical education, providing the spatial understanding of organ systems upon which clinical reasoning and procedural competency are built [1]. Traditional cadaveric dissection is the pedagogical cornerstone of anatomy instruction; however, growing ethical, logistical, and resource constraints are increasingly driving the adoption of complementary digital platforms [2, 3]. Meta-analytic evidence confirms that laboratory-based anatomical education—including technology-enhanced approaches—significantly improves student knowledge outcomes compared to didactic instruction alone [4]. 

Digital anatomy is emerging as a new discipline that is rapidly transforming how anatomical structures are taught and visualized [5]. A scoping review of 156 studies across six health disciplines found that three-dimensional digital tools, augmented reality, and virtual reality are the most commonly adopted technologies for anatomy education globally [6]. Meta-analytic evidence further demonstrates that virtual reality-based anatomy instruction produces moderate improvements in examination scores compared to conventional two-dimensional methods (standardized mean difference = 0.53) [7]. Recent reviews confirm that digital technologies substantially enhance engagement, spatial understanding, and student satisfaction in undergraduate anatomy courses [8]. 

The Anatomage virtual dissection table (Anatomage Inc., San Jose, CA) is among the most sophisticated platforms for technology-enhanced anatomy teaching, offering interactive virtual dissection, annotation of thousands of structures, Digital Imaging and Communications in Medicine (DICOM)-compatible medical imaging integration, ultra-high-quality (UHQ) volumetric rendering, and quiz mode functionality [9, 10]. Published evidence consistently demonstrates favorable student perspectives toward the Anatomage for three-dimensional anatomical visualization, supporting its use as a valued complement to cadaveric dissection rather than a replacement [10–13]. 

A critical but underappreciated limitation of the Anatomage table is its scalability. In conventional deployment, the device is used by small groups of 6 to 15 students gathered around its 84-inch touchscreen surface in the dissection laboratory. A medical college enrolling more than 100 students per batch cannot simultaneously deliver Anatomage-based teaching to the entire cohort from a single unit, and the high acquisition cost makes procurement of multiple units impractical for most institutions [10]. This scalability gap represents a significant barrier to equitable access to technology-enhanced anatomy education, particularly in institutions in lower-resource settings [5, 6]. 

Remote streaming of the live Anatomage interface to a lecture theatre via a smart digital podium offers a practical solution to this scalability constraint. A podium equipped with a touchscreen, institutional LAN connectivity, and High-Definition Multimedia Interface (HDMI) output can access the Anatomage table over the network and project its interactive interface onto dual classroom screens, enabling a single faculty member to deliver Anatomage-integrated anatomy teaching to an entire cohort simultaneously. This multimodal approach aligns with Mayer’s cognitive theory of multimedia learning, which predicts that combining verbal explanation with rich spatial imagery produces deeper memory encoding than verbal-only instruction [14]. 

Neuroanatomy represents a particularly challenging pedagogical domain for first-year medical students. The basal nuclei—comprising the caudate nucleus, putamen, globus pallidus, subthalamic nucleus, and substantia nigra—form a functionally critical subcortical circuit whose three-dimensional inter-relationships are poorly conveyed by two-dimensional diagrams [15]. Blended approaches integrating digital and traditional modalities have shown promise in the Indian medical undergraduate context [16, 17]. 

The COVID-19 pandemic accelerated experimentation with remote anatomy delivery, including synchronous online dissection via videoconferencing platforms and mixed-reality tools such as HoloLens-based HoloAnatomy; however, these approaches streamed content to students’ personal devices in geographically dispersed settings rather than projecting a shared live interactive interface within a physical lecture hall [5, 8]. The RAT model described here is architecturally distinct: it uses an institutional LAN to stream the full Anatomage interface to a single shared display space, preserving the social and pedagogical dynamics of in-person large-group teaching while extending access to a sophisticated virtual anatomy platform. To our knowledge, no prior study has evaluated the feasibility or student acceptability of this LAN-based large-group streaming model. The present study is accordingly framed as a preliminary proof-of-concept investigation. It describes the technical implementation of RAT and reports initial student perspectives following its first large-group deployment for a basal nuclei lecture at Kasturba Medical College, Manipal Academy of Higher Education, Manipal, India. Definitive conclusions about educational efficacy will require repeated implementation across multiple sessions, topics, student cohorts, and institutions with objective learning outcome measures; the present findings are intended to establish feasibility and generate effect-size estimates needed to power a formal controlled trial.

## Methods

### Study design, setting, and ethical approval

This cross-sectional descriptive study was conducted in the Department of Anatomy, Kasturba Medical College, Manipal Academy of Higher Education, Manipal, India (academic year 2024–2025). The study is reported in accordance with the STROBE (Strengthening the Reporting of Observational Studies in Epidemiology) guideline for cross-sectional studies; a completed STROBE checklist is provided as Additional file 3. The electronic survey component additionally followed the principles of the CHERRIES (Checklist for Reporting Results of Internet E-Surveys) guideline. The study was approved by the Institutional Ethics Committee (IEC; Reference: IEC 14/2025). All procedures comply with the World Medical Association Declaration of Helsinki. Participation in the post-session questionnaire was voluntary, anonymous, and carried no academic consequences. Written informed consent was obtained from all participants. Informed consent for classroom photography and video recording was obtained from all students and faculty visible in the reproduced materials.

### Participants

All 125 first-year medical students who attended the integrated basal nuclei lecture were eligible to participate. A cross-sectional survey design was employed, with the questionnaire administered immediately after the teaching session as the primary data collection instrument. Of the 125 attendees, 94 completed the post-session questionnaire (response rate: 75.2%, 94/125). The remaining 31 students did not complete the questionnaire, these students remained present for the full session and chose not to complete the optional, anonymous form, most plausibly reflecting unwillingness to spend additional time on a non-mandatory survey immediately following a 47-minute lecture, though early departure or technical access difficulty cannot be excluded as alternative explanations.

### The Remote Anatomage Teaching (RAT) setup

#### Overview

The RAT model comprises three integrated components that together stream the full interactivity of the Anatomage table in real time to a large lecture theatre without requiring either students or the instructor to be physically present at the table.

#### Component 1: anatomage table

The institution operates one Anatomage Table software version 11.0 (Convertible) installed in the dissection laboratory, featuring an 84-inch touchscreen display, UHQ volumetric rendering, interactive dissection and annotation tools, DICOM imaging integration, and quiz mode [10]. For a RAT session, the table is powered and maintained in its active state in the dissection laboratory throughout the lecture; no dedicated operator is required at the table.

#### Component 2: smart digital podium

A smart digital podium (Evolved Axis Private Limited [EAPL Technologies], New Delhi, India) installed in the lecture theatre incorporates a 21.5-inch multi-touch motorized display (1920 × 1080 pixels, up to 60° motorized tilt), Core i5 computer (16 GB RAM, 512 GB SSD), gooseneck microphone, and HDMI video output. The Anatomage table interface is accessed on the podium computer via a dedicated, low-latency institutional LAN segment rather than shared internet bandwidth, replicating full interactivity—structure isolation, rotation, layered dissection, annotation, and zoom—without requiring the instructor to be physically present at the table; this dedicated network configuration is believed to have contributed to the absence of connectivity lag observed during the session. The podium’s HDMI output is connected to dual ceiling-mounted projectors, simultaneously projecting the live Anatomage interface on two large screens flanking the central blackboard.

#### Component 3: multimodal lecture delivery

**Fig. 1:**
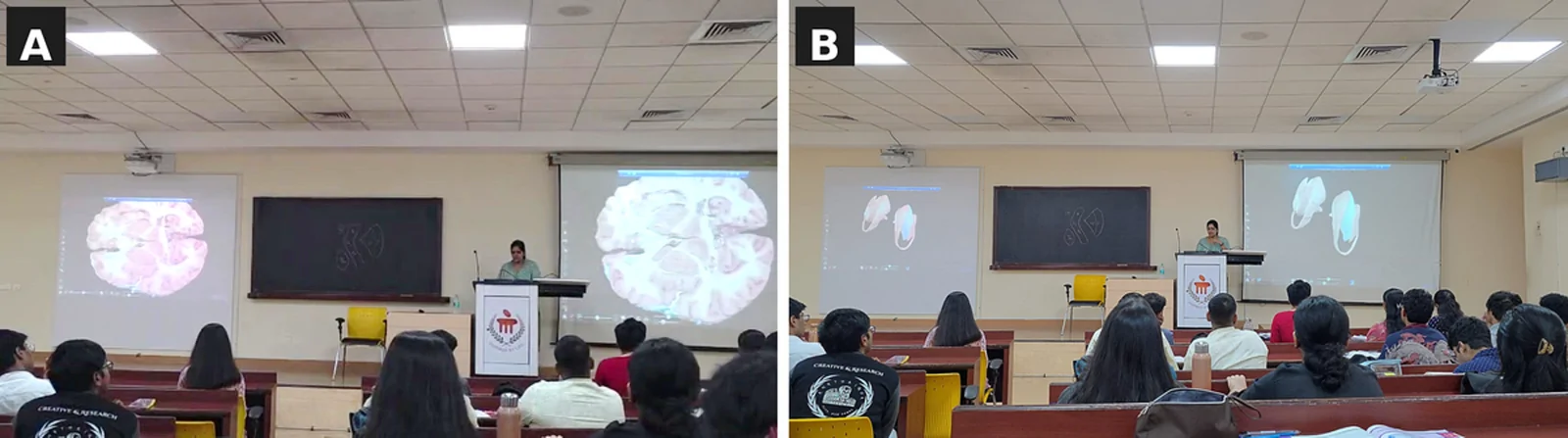
Remote Anatomage Teaching (RAT) session. **A** Lecture theatre overview showing an Anatomage transverse (axial) brain section through the basal nuclei projected on both screens via the smart digital podium, remotely connected over the institutional LAN to the Anatomage Table software version 11.0 in the dissection laboratory. **B** Faculty member operating the smart digital podium; a three-dimensional volumetric rendering of the basal nuclei is visible on both projector screens. Both panels (**A**) and (**B**) show Anatomage-generated visualizations. Photographs reproduced with informed participant consent. EAPL: Evolved Axis Private Limited; LAN: local area network; RAT: Remote Anatomage Teaching

The faculty member operated the RAT session entirely from the podium touchscreen, integrating three complementary modalities: (1) structured blackboard diagrams for circuit-level explanation of cortico-striato-pallido-thalamo-cortical pathways; (2) PowerPoint slides for clinical correlations (Parkinson disease, Huntington disease, and hemiballismus); and (3) live remote Anatomage navigation for three-dimensional visualization of the caudate nucleus, putamen, globus pallidus, subthalamic nucleus, and substantia nigra and their inter-relationships with the internal capsule and thalamus. The lecture (47 min) was delivered to all 125 first-year medical students. The classroom setup is illustrated in Fig. 1, and a representative video recording is provided as Additional file 1.

### Attitude questionnaire

Immediately following the lecture, students completed a 13-item attitude questionnaire administered anonymously via Microsoft Forms (Microsoft Corporation; mean completion time: 1 min 31 s); the complete English-language version is provided as Additional file 2. The questionnaire was developed de novo by the research team; no items were reproduced from prior published instruments, but the domain structure was informed by two published Anatomage attitude questionnaires [12, 13] to ensure content relevance and comparability with prior literature. It was not independently validated as a new psychometric instrument. Content validity was established by independent review by two senior anatomy faculty members prior to administration, and face validity was confirmed in a pilot run with five students from a separate batch not included in the study sample.

The questionnaire comprised 13 items: Item 1 (gender, collected for participant characterization purposes only and not used analytically in any reported comparison); Item 2 (engagement, 5-point Likert scale comparing RAT to regular theory classes, from much more engaging to much less engaging); Item 3 (understanding), Item 4 (skill development), Item 6 (comfort level), Item 7 (collaboration), Item 8 (long-term retention), Item 9 (instructor feedback), and Item 10 (self-paced learning): seven comparative forced-choice items (response options: RAT, PowerPoint-based lecture, or both equally/no difference); Item 5 (realism of the Anatomage compared to cadaveric dissection, 5-point Likert scale); Item 11 (interest in anatomy, 4-point Likert scale from much more interested to relatively less interested); Item 12 (curriculum integration, 5-point Likert scale from strongly support to strongly oppose); and Item 13 (one open-ended item inviting free-text opinions).

### Statistical analysis

This is a descriptive cross-sectional study reporting frequencies and percentages. No inferential statistics were performed given the single-arm, single-session design. Internal consistency of nine attitudinal preference items (Items 3, 4, 6, 7, 8, 9, 10, 11, and 12: the seven comparative items, Interest in Anatomy, and Curriculum Integration, excluding the Realism item [Item 5] which measures a conceptually distinct construct) was assessed post hoc using Cronbach’s alpha. For this computation, the seven comparative items were numerically coded as RAT = 3, Both equally/No difference = 2, Regular PPT-based lecture = 1; the Interest in Anatomy item was coded 4 (much more interested) to 1 (relatively less interested); and the Curriculum Integration item was coded 5 (strongly support) to 1 (strongly oppose); all items were oriented so that higher scores reflected greater preference for RAT. This yielded α = 0.873 (*n* = 94). Alpha is used here as a descriptive baseline estimate of response consistency across nine conceptually distinct but directionally aligned preference items, rather than as confirmation of a unidimensional psychometric construct; we caution that a high alpha across structurally different pedagogical domains may reflect response-style consistency rather than true latent unidimensionality, and the questionnaire should not be interpreted as a formally validated psychometric scale.

Free-text responses underwent reflexive thematic analysis following Braun and Clarke’s [18] six-phase framework. Two investigators independently familiarised themselves with and coded all retained substantive responses, generated candidate themes independently, then met to compare codes and discuss discrepancies until full consensus was reached on the final themes; no third arbitrator was required, as full consensus was achieved without need for escalation. No formal inter-rater reliability statistic (e.g., Cohen’s kappa) was computed, which we acknowledge as a limitation. Of 94 respondents, 87 provided a non-blank entry to the open-ended item (7 left it blank). A priori exclusion criteria—defined before coding began—classified an entry as non-informative if it consisted of: (i) a single-word null-content response (e.g., “none,” “no,” “nil,” “nothing”); (ii) a generic, unelaborated remark with no specific reference to either teaching modality (e.g., “good,” “better,” “all good”); (iii) a punctuation- or symbol-only entry (e.g., “-”, “.”); or (iv) an entry of two characters or fewer with no discernible content. Applying these criteria, 57 of the 87 non-blank entries were classified as non-informative, leaving 30 substantive responses for thematic analysis. Thematic saturation was not formally assessed given the small number of substantive responses; the three themes identified should be regarded as the principal recurring patterns observed within this preliminary sample rather than a confirmed saturated thematic landscape.

## Results

### Technical feasibility

The RAT session was delivered successfully without technical interruption. The Anatomage Table maintained a stable LAN connection to the smart digital podium throughout the 47-minute lecture. Full interactivity—three-dimensional rotation, layered dissection revealing the internal capsule and lentiform nucleus, zoom on the subthalamic nucleus, and blood flow animation in the striatal vasculature—was available to the faculty at the podium and projected in real time on both lecture theatre screens. No connectivity failure, image freezing, or session discontinuity was recorded. The technical setup was established within 10 min at the start of the session.

### Participant characteristics

Of 125 students who attended the lecture, 94 completed the questionnaire (response rate: 75.2%, 94/125); the remaining 31 students did not complete the form, for reasons that could not be independently confirmed (see Methods, Participants). The 94-respondent sample comprised 54 females, 37 males, and 3 who preferred not to disclose gender.

### Student engagement

Thirty-six out of 94 students (38.3%) rated RAT as ‘much more engaging’ and 36/94 (38.3%) as ‘more engaging’ than regular theory classes, giving a combined positive engagement response of 72/94 (76.6%). A further 17/94 (18.1%) found it ‘about the same’ and 5/94 (5.3%) ‘less engaging.’ No student (0/94) rated RAT as ‘much less engaging.’

### Comparative attitudes across pedagogical domains

**Fig. 2:**
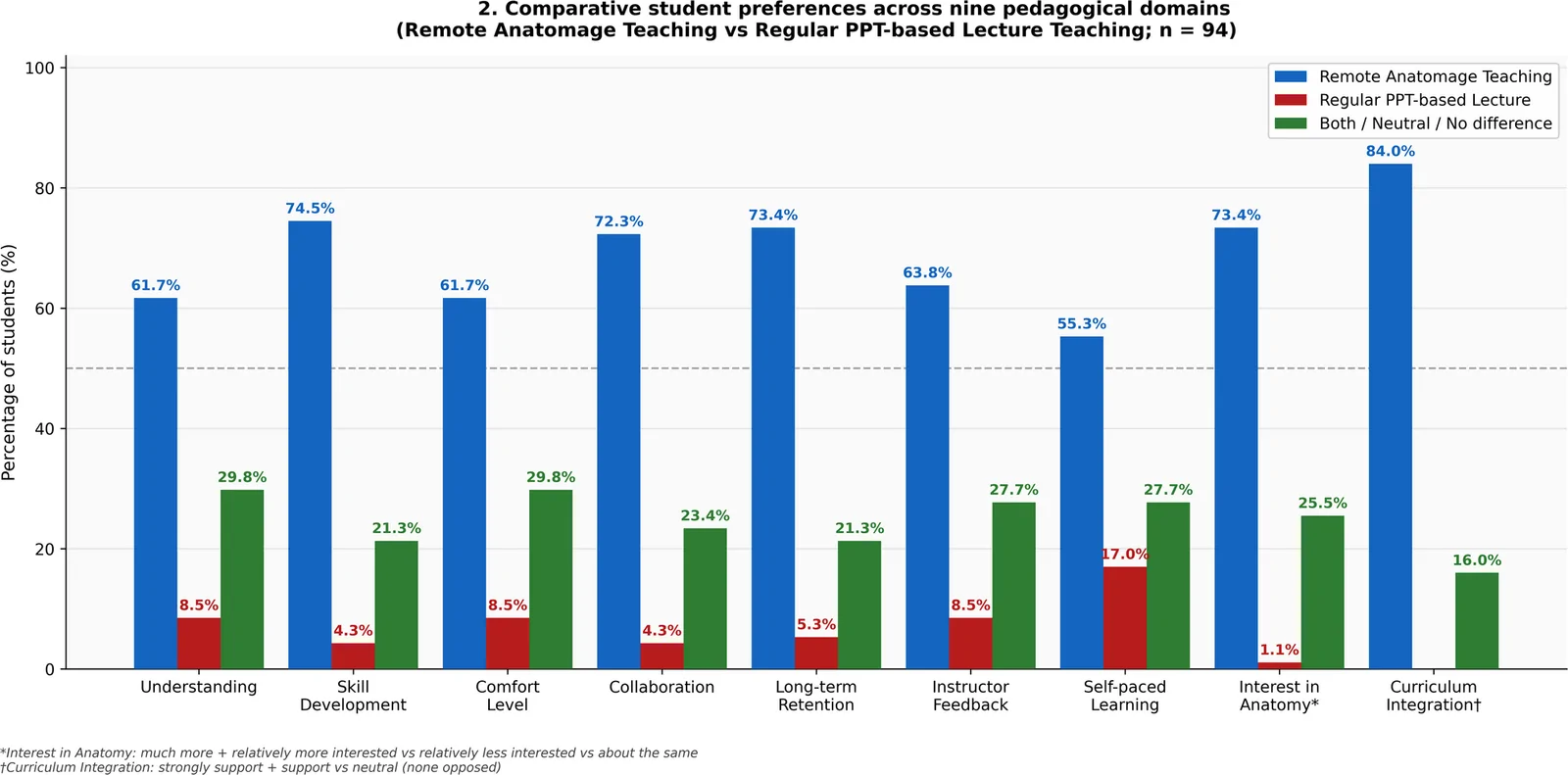
Grouped bar chart comparing student preferences for Remote Anatomage Teaching (RAT), regular PowerPoint-based lecture teaching, and both/neutral across nine pedagogical domains (*N* = 94). The dashed reference line at 50% is exceeded in all nine domains. *Interest in Anatomy: response categories ‘much more interested’ and ‘relatively more interested’ combined vs. ‘relatively less interested’ vs. ‘about the same.’ †Curriculum Integration: response categories ‘strongly support’ and ‘support’ combined vs. ‘neutral’ (no students selected ‘oppose’ or ‘strongly oppose’). PPT: PowerPoint; RAT: Remote Anatomage Teaching

RAT was preferred over PowerPoint-based lecture teaching in every domain, with preferences ranging from 55.3% (52/94) for self-paced learning to 84.0% (79/94) for curriculum integration. Curriculum integration was endorsed (strongly support or support) by 79/94 students (84.0%), with no students opposing integration. Figure 2 summarizes the comparative preferences across all nine domains. The dashed reference line at 50% is exceeded in all nine domains.

### Realism, interest in anatomy, and curriculum integration

**Fig. 3:**
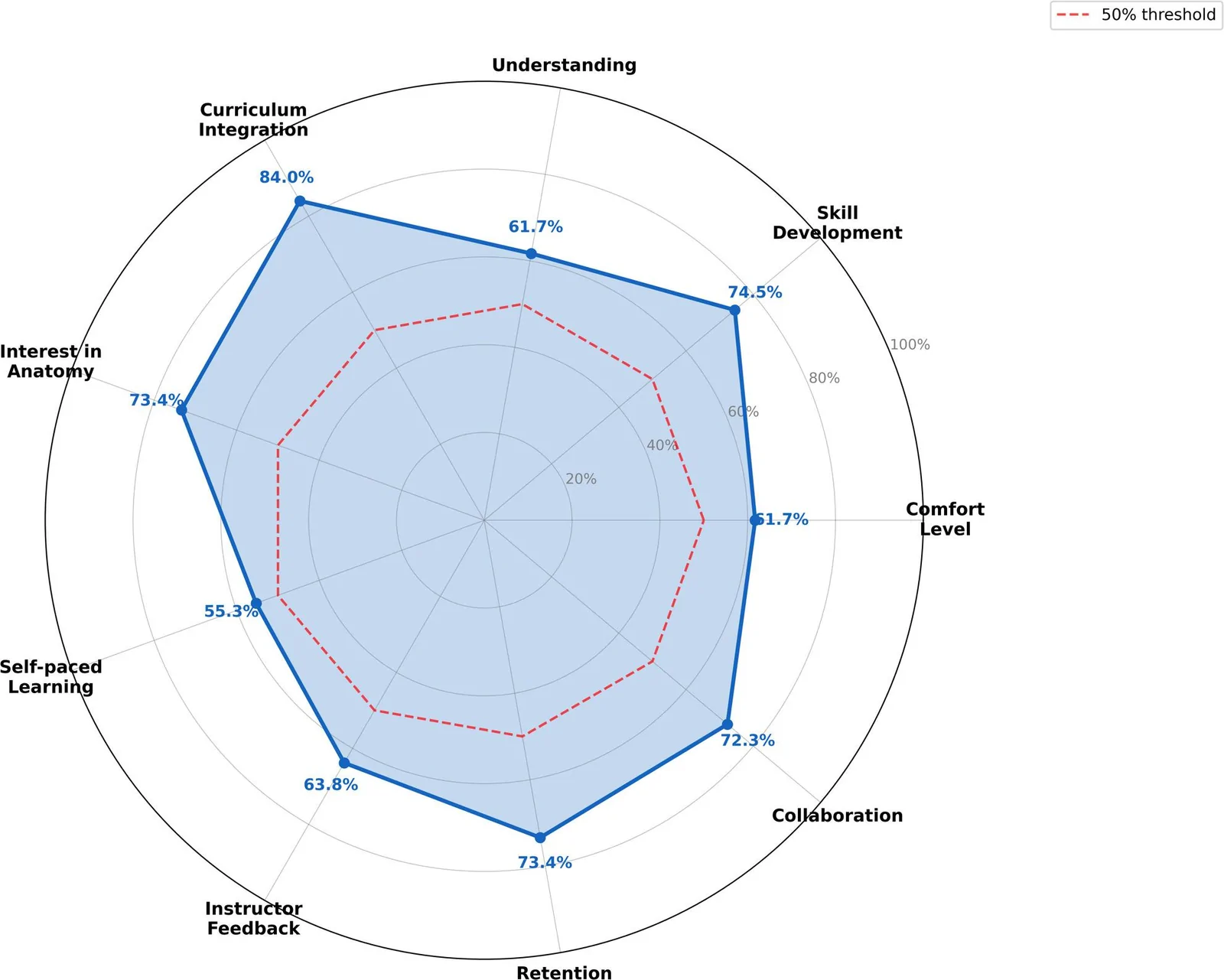
Radar chart of the percentage of students preferring Remote Anatomage Teaching (RAT) across nine pedagogical domains (*N* = 94). Curriculum Integration is included as the ninth domain; its plotted percentage represents the combined proportion of ‘strongly support’ and ‘support’ responses, analogous to the treatment of the eight comparative forced-choice and Likert preference domains. The dashed red circle marks the 50% reference threshold; all nine domains exceed this threshold. RAT: Remote Anatomage Teaching

Of 94 students, 14 (14.9%) rated the Anatomage as ‘very realistic’ and 63 (67.0%) as ‘somewhat realistic’ compared to actual cadaveric dissection, giving a combined realism endorsement of 77/94 (81.9%). Nine out of 94 (9.6%) were neutral and 8/94 (8.5%) found it ‘somewhat unrealistic’; no student found it ‘very unrealistic.’ Interest in anatomy was reported as increased by 73.4% of respondents (69/94), combining the ‘much more interested’ and ‘relatively more interested’ response categories of Item 11. Figure 3 presents a radar chart of the proportion preferring RAT across all nine comparative domains, with the highest preferences for curriculum integration (84.0%, 79/94) and skill development (74.5%, 70/94), and the lowest for self-paced learning (55.3%, 52/94).

### Qualitative findings

Of 94 respondents, 87 provided a non-blank entry to the open-ended item; applying the a priori exclusion criteria described in Methods, 57 entries were classified as non-informative, leaving 30 substantive responses for reflexive thematic analysis. Three recurring themes emerged with full consensus between the two investigators. The first theme—*visual engagement and retention*—was most prominent: students described RAT as ‘more interactive,’ superior for ‘visual retention,’ and explicitly helpful in making structures ‘visible’ and ‘easier to understand.’ The second theme—*preference for blended integration*—reflected pragmatic advocacy: students suggested combining both modalities ‘would be very effective,’ with PowerPoint-based teaching valued for clinical case discussion and the Anatomage preferred for three-dimensional structural visualization. The third theme—*projection visibility*—was raised by 2/94 students (2.1%) who found structures ‘not clearly visible’ when projected at distance through standard classroom projectors, identifying the primary technical constraint of the current implementation.

## Discussion

### Principal findings

This cross-sectional study introduces Remote Anatomage Teaching and confirms that large-group virtual anatomy delivery via a smart digital podium in a lecture hall is technically feasible and elicits strongly favorable student perspectives across all measured pedagogical domains. To our knowledge, this is the first published report of the Anatomage table being used in a fully remote streaming configuration to teach an entire medical batch simultaneously in a large lecture hall. The Anatomage Table streamed its interactive interface stably over the institutional LAN throughout the 47-minute lecture without connectivity failure, with full three-dimensional interactivity preserved and projected in real time on dual classroom screens—a meaningful departure from all prior descriptions of Anatomage use, which have exclusively described small-group, table-side delivery [9–13]. 

Among respondents, 72/94 (76.6%) rated RAT as more engaging or much more engaging than regular theory classes. These findings are consistent with prior studies of conventional small-group Anatomage delivery: Alasmari reported that approximately 89% of medical students agreed the Anatomage helped them understand anatomical relationships [12], and Baratz et al. found positive student perspectives in a crossover cadaveric-virtual design [13]. That a remote, projected delivery model preserves these favorable perspectives is a meaningful finding, extending the evidence base for digital anatomy tools to a large-group lecture hall context [5, 6]. As Fyfe et al. noted, student ratings of the Anatomage improve with greater familiarity [11], suggesting our single-session findings represent a conservative estimate of the model’s acceptability. It is important to acknowledge that the consistently positive response pattern may partly reflect novelty bias inherent to single-session evaluations of unfamiliar technologies; students encountering a novel, visually engaging tool for the first time tend to rate it favourably irrespective of its learning efficacy. We also note a distinct, related concern: the questionnaire was administered in the immediate post-session classroom environment, in physical proximity to the teaching faculty, which may have introduced social desirability bias toward favourable responses despite the anonymous electronic format. However, two observations partly mitigate the novelty-bias concern. First, the preference for RAT was consistent across all nine independent pedagogical domains—including self-paced learning (55.3%), a domain where a projected shared interface is structurally less suited than individual access—suggesting genuine domain-specific judgements rather than global positivity bias. Second, the absence of any opposition to curriculum integration (0/94) provides convergent evidence that students were making thoughtful pedagogical assessments. That said, repeated-session data across multiple topics and cohorts, ideally incorporating an active comparator condition, will be necessary to determine whether perspectives are sustained once novelty diminishes and to disentangle genuine RAT-specific benefit from a generic novelty-of-addition effect that might accompany any enhancement to a standard didactic lecture.

The preference for RAT in skill development (74.5%, 70/94) and retention (73.4%, 69/94) is consistent with Mayer’s cognitive theory of multimedia learning [14], which predicts that integrating verbal explanation with rich spatial imagery produces deeper memory encoding than verbal-only formats—directly relevant to anatomy, where spatial mastery is fundamental [19]. Meta-analytic evidence from virtual reality-based anatomy interventions confirms that three-dimensional digital tools produce moderate to large improvements in examination scores compared to conventional two-dimensional instruction [7]. The basal nuclei—whose complex subcortical inter-relationships are poorly conveyed in two-dimensional diagrams [15]—represent an ideal application of this principle. We note, however, that first-year students, who have not yet entered clinical rotations, may lack an adequate frame of reference for self-assessing “skills needed for their future career,” and the high RAT preference observed in this domain may reflect general enthusiasm for novel technology rather than a verified judgement of clinical skill utility; this is addressed further in Limitations.

Curriculum integration was endorsed by 79/94 students (84.0%) with no opposition, mirroring findings from blended anatomy programs globally. Darras et al. demonstrated that first-year medical students perceived enhanced learning when virtual dissection was combined with cadaveric laboratory work [16], and Sarkar et al. reported high satisfaction with blended anatomy delivery in an Indian MBBS cohort directly comparable to ours [17]. Our qualitative data show complementary preferences: the Anatomage for three-dimensional structural visualization and PowerPoint-based teaching for clinical case discussion. This division of pedagogical labour aligns with the Competency-Based Medical Education (CBME) philosophy promoted by the National Medical Commission (NMC), whose 2024 CBME Curriculum guidelines advocate diverse pedagogical approaches to achieve measurable competencies [20], and with the broader trend in digital anatomy education toward multimodal, learner-centred integration [5, 6, 8]. 

A question naturally arises as to why a live-streamed interactive interface was used rather than a pre-recorded video of the same Anatomage content, or a freely available web-based three-dimensional anatomy application. Unlike a pre-recorded video, live streaming preserves the instructor’s ability to respond in real time to student questions—rotating, isolating, or re-dissecting structures on demand—replicating the pedagogical responsiveness of table-side teaching rather than offering a fixed, non-interactive viewing experience. Freely available web-based three-dimensional anatomy models, while valuable, are typically built from idealised or schematic representations rather than the Anatomage’s cadaver-derived UHQ volumetric datasets, and do not leverage an institution’s existing dissection laboratory infrastructure and Anatomage investment. RAT was conceived specifically to extend the reach of an institution’s own high-fidelity asset rather than to substitute it with a generic alternative.

RAT was delivered in this study as a large-group lecture-hall supplement alongside, not a replacement for, the institution’s unchanged small-group cadaveric dissection curriculum, which continues to run in parallel laboratory sessions. We acknowledge a theoretical risk that enthusiasm for large-group technology-enhanced teaching could, in principle, reduce student motivation for hands-on dissection; this possibility was not assessed in the present study and is identified as a specific outcome measure for the planned controlled trial.

The realism endorsement of 81.9% (77/94), achieved in the remote projected format, reflects the fidelity of the Anatomage’s UHQ volumetric rendering at lecture-hall scale. Owolabi et al. identified anatomical accuracy and annotative richness as the most valued educator-facing attributes of the Anatomage globally; [21] our student-facing data suggest these qualities are perceived as preserved even when viewed via projection rather than direct table-side interaction. The higher endorsement of ‘somewhat realistic’ (67.0%) over ‘very realistic’ (14.9%) reflects the inherent limitations of digital reconstruction—absence of tactile and olfactory cues—that persist regardless of delivery modality and reinforce why cadaveric dissection cannot be entirely replaced [4]. 

From an implementation standpoint, the RAT model addresses a genuine scalability barrier that currently limits the reach of the Anatomage in most institutions. Remote streaming enables a single installed Anatomage unit to serve large-group lecture teaching, multiplying the educational reach of a substantial capital investment without additional hardware procurement. For Indian medical colleges under NMC oversight, this represents a cost-effective pathway to expand technology-enhanced anatomy instruction in alignment with CBME requirements.

The projection visibility concern raised by 2/94 students (2.1%) identifies the primary technical limitation of the current implementation. Standard classroom projectors introduce resolution loss and ambient light interference at distance, particularly for fine structures such as the subthalamic nucleus and individual fibre tracts. Planned technical improvements include high-lumen projectors (≥ 5,000 ANSI lumens), full ambient light control, and supplementary close-up zooming on the podium touchscreen before projection.

### Limitations

This preliminary proof-of-concept study has its limitations. The single post-session cross-sectional design does not permit causal inferences about learning outcomes; we report student perspectives and technical feasibility, not objective knowledge gains. The absence of an active comparator condition—such as a small-group interactive session or a freely available three-dimensional anatomy application—means the favourable perspectives reported here cannot be distinguished from a generic novelty-of-addition effect that might accompany any enhancement to a standard didactic lecture, irrespective of its specific pedagogical content. Findings are from a single institution, a single student batch, and a single neuroanatomy topic, limiting generalizability; RAT may perform differently for topics with lower three-dimensional complexity or for cohorts with prior Anatomage familiarity. Novelty bias cannot be entirely excluded, though consistency of preferences across structurally dissimilar domains mitigates this concern; additionally, administration of the questionnaire in the immediate post-session classroom environment, in proximity to the teaching faculty, may have introduced social desirability bias despite the anonymous electronic format. First-year students’ self-assessment of “skills needed for their future career” (Item 4) is constrained by their lack of clinical rotation experience and may capture general technological enthusiasm rather than verified educational utility. One item differed in response format from the others: Item 6 (Comfort Level) offered four options (RAT / Regular PPT / Both equally / No difference) rather than the three options of the other comparative items; for analysis, ‘No difference’ was treated as equivalent to ‘Both equally’ and merged into the neutral category, as documented in Additional file 2. While the questionnaire demonstrated good internal consistency on a descriptive basis (α = 0.873), it was not formally validated as a new psychometric instrument, and construct validity evidence was not generated. The 24.8% non-response rate (31/125) introduces a recognised risk of response bias toward students with more favourable prior attitudes toward novel technology, since the characteristics and perspectives of non-responders are, by definition, unknown; we could not confirm whether non-response reflected voluntary opt-out, early departure, or technical access difficulty. The dual-investigator qualitative consensus process did not include a formal inter-rater reliability statistic (e.g., Cohen’s kappa), and thematic saturation was not formally assessed given the small substantive sample (*n* = 30). No long-term retention or objective examination data are reported, which are essential before any claims about educational efficacy can be made.

### Future directions

The critical next step is a formal randomized controlled trial comparing objective learning outcomes between students receiving RAT and those receiving conventional PowerPoint-based instruction, incorporating an active comparator arm (e.g., a small-group interactive session or a freely available three-dimensional anatomy application) rather than a passive PowerPoint-only control, directly addressing the absence of an active comparator in this preliminary study. As a preliminary proof-of-concept study, the present findings cannot be taken as evidence of educational efficacy; they establish technical feasibility and generate effect-size and acceptability estimates needed to power and justify such a trial [7]. Before the controlled trial is conducted, RAT should be implemented across multiple topics and student cohorts within the same institution to assess whether the strongly favourable perspectives reported here are sustained beyond a single novel encounter. Planned outcome measures will include immediate post-session knowledge tests, delayed retention assessments at four to six weeks, spatial reasoning assessments for basal nuclei constructs, and a measure of engagement with concurrent cadaveric dissection sessions to assess whether large-group technology-enhanced teaching affects motivation for hands-on dissection. Future iterations should incorporate high-lumen classroom projectors (≥ 5,000 ANSI lumens) and full ambient light control to address projection visibility, and should systematically document setup time, connection reliability, and projection quality as process outcomes. Broader, multi-institutional implementation—particularly in lower-resource settings where a single Anatomage unit must serve large cohorts [5]—would test generalizability. On a curricular level, RAT could be paired with small-group Anatomage laboratory sessions to create a tiered blended learning model, aligning with best-practice frameworks for technology-enhanced anatomy education [6, 8]. 

## Conclusions

Large-group Remote Anatomage Teaching via a smart digital podium is technically feasible and elicits strongly favorable student perspectives across multiple pedagogical domains, including skill development, long-term retention, and curriculum integration. This scalable model extends virtual anatomy technology to large lecture hall settings without additional hardware investment, serving as a cost-effective complement to cadaveric and conventional lecture-based teaching. A powered randomized controlled trial incorporating an active comparator arm and comparing objective learning outcomes is warranted as the definitive next step.

## Supplementary Information

Supplementary Material 1: Additional file 1. Supplementary Video 1. A 69-second video recording of the Remote Anatomage Teaching (RAT) session demonstrating live three-dimensional navigation of the basal nuclei projected on dual classroom screens via the smart digital podium. Full interactivity including structure rotation, layered dissection, and blood flow animation is visible. Informed participant consent for recording and reproduction was obtained from all visible students and faculty. [MP4 file]

Supplementary Material 2: Additional file 2. Attitude Questionnaire on Anatomy Teaching Methods: Remote Anatomage Teaching vs Regular PPT-Based Lecture Teaching. Complete English-language version of the 13-item questionnaire developed for this study, including all item wording, response options, item format, administration details, ethics statement, and notes on response option labelling. [DOCX file]

Supplementary Material 3: Additional file 3. Completed STROBE (Strengthening the Reporting of Observational Studies in Epidemiology) checklist for cross-sectional studies, mapping all applicable items to their corresponding locations in this manuscript. [DOCX file]

## Data Availability

The anonymized dataset supporting the conclusions of this article is available from the corresponding author on reasonable request. Individual survey responses are not publicly available to protect participant anonymity given the identifiable cohort size.

## Abbreviations

CBME: Competency-based Medical Education
CHERRIES: Checklist for Reporting Results of Internet E-Surveys
DICOM: Digital Imaging and Communications in Medicine
EAPL: Evolved Axis Private Limited
HDMI: High-Definition Multimedia Interface
IEC: Institutional Ethics Committee
LAN: local Area Network
MAHE: Manipal Academy of Higher Education
NMC: National Medical Commission
PPT: Power Point
RAT: Remote Anatomage Teaching
STROBE: Strengthening the Reporting of Observational Studies in Epidemiology
UHQ: Ultra-high Quality
