# Supplementary material for: Large-group virtual anatomy via a smart digital podium: a preliminary cross-sectional study of student perspectives toward remote anatomage teaching in a medical lecture hall
Source: BMC Med Educ. 2026 Jul 25;26:1684. doi: 10.1186/s12909-026-10019-x (PMC13685385; doi:10.1186/s12909-026-10019-x)
Supplement: Supplementary file 1 [file 12909_2026_10019_MOESM2_ESM.docx]

**Additional file 2**

**Attitude Questionnaire on Anatomy Teaching Methods**

*Remote Anatomage Teaching vs Regular PPT-Based Lecture Teaching*

Department of Anatomy, Kasturba Medical College, Manipal Academy of Higher Education, Manipal, India

Academic year 2024–2025 | First-year MBBS students

Date of administration: January 6, 2025 | Platform: Microsoft Forms

**Ethics and consent:** This questionnaire was approved by the Institutional Ethics Committee (IEC) of Manipal Academy of Higher Education (Reference: IEC 14/2025). Participation was entirely voluntary, anonymous, and carried no academic consequences. Written informed consent was obtained from all participants prior to administration.

**Instructions to participants:** You have just attended a basal nuclei lecture delivered using Remote Anatomage Teaching (RAT) via the smart digital podium. Please answer all items honestly based on your experience of this session. There are no right or wrong answers.

**Questionnaire development:** This questionnaire was developed de novo by the research team for this study. The domain structure was informed by published Anatomage attitude instruments [Alasmari WA. Adv Med Educ Pract. 2021;12:1303–1307; Baratz G et al. Med Sci Educ. 2019;29(2):499–506] to ensure content relevance and comparability with prior literature. The questionnaire was administered electronically via Microsoft Forms immediately post-session; mean completion time was 1 minute 31 seconds. Internal consistency of nine attitudinal preference items (Items 3, 4, 6–12, excluding Realism [Item 5]) yielded Cronbach’s α = 0.873 (n = 94).

| **Item** | **Domain** | **Question** | **Response options** | **Format** |
| --- | --- | --- | --- | --- |
| 1 | Participant information | What is your gender? | Female Male Prefer not to say | Single-choice |
| 2 | Engagement (primary outcome) | How engaging do you find Remote Anatomage Teaching compared to a regular theory class? | Much more engaging More engaging About the same Less engaging Much less engaging | 5-point Likert (engagement) |
| 3 | Understanding | Which method enhances your understanding of the anatomy topic more effectively? | Remote Anatomage Teaching Regular PPT-based lecture teaching Both equally | 3-option comparative forced-choice |
| 4 | Skill development | Which method do you believe better develops the practical skills needed for your future career? | Remote Anatomage Teaching Regular PPT-based lecture teaching Both equally | 3-option comparative forced-choice |
| 5 | Realism | How realistic do you find the Anatomage compared to an actual dissected cadaver? | Very realistic Somewhat realistic Neither realistic nor unrealistic Somewhat unrealistic Very unrealistic | 5-point Likert (realism) |
| 6 | Comfort level | Which method makes you feel more comfortable learning anatomy? | Remote Anatomage Teaching Regular PPT-based lecture teaching Both equally No difference | 4-option comparative forced-choice |
| 7 | Collaboration | Which method promotes better collaboration and interaction among students and teacher? | Remote Anatomage Teaching Regular PPT-based lecture teaching Both equally | 3-option comparative forced-choice |
| 8 | Long-term retention | Which method do you think leads to better long-term retention of anatomical knowledge? | Remote Anatomage Teaching Regular PPT-based lecture teaching Both equally | 3-option comparative forced-choice |
| 9 | Instructor feedback | Which method allows for better feedback from instructors? | Remote Anatomage Teaching Regular PPT-based lecture teaching Both equally | 3-option comparative forced-choice |
| 10 | Self-paced learning | Which method allows you to learn at your own pace more effectively? | Remote Anatomage Teaching Regular PPT-based lecture teaching Both equally | 3-option comparative forced-choice |
| 11 | Interest in anatomy | Has your interest in anatomy increased through Anatomage Teaching compared to a regular PPT-based lecture? | Much more interested Relatively more interested About the same Relatively less interested | 4-point Likert (interest) |
| 12 | Curriculum integration | Would you support a curriculum that integrates both Anatomage and regular PPT-based lecture teaching for a comprehensive anatomy education? | Strongly support Support Neutral Oppose Strongly oppose | 5-point Likert (support) |
| 13 | Open-ended | Any suggestions or opinions regarding Regular PPT-based lecture teaching vs Anatomage Teaching? | Free-text response | Open-ended |

**Notes on response options:**

(a) Items 3, 4, and 6–10: comparative forced-choice items comparing Remote Anatomage Teaching (RAT) vs Regular PPT-based lecture teaching. Item 5 (Realism) is a separate 5-point Likert scale item and is not a comparative forced-choice item; it was excluded from the Cronbach’s α computation. comparing Remote Anatomage Teaching (RAT) vs Regular PPT-based lecture teaching. "Both equally" and "No difference" treated as equivalent neutral responses in analysis.

(b) Item 12 (Curriculum Integration): Response options "Oppose" and "Strongly oppose" appeared in the form but were selected by 0 respondents.

(d) Item 2 (Engagement): Response option "Much less engaging" appeared in the form but was selected by 0 respondents.

(e) Item 5 (Realism): Response option "Very unrealistic" appeared in the form but was selected by 0 respondents.

*PPT: PowerPoint; RAT: Remote Anatomage Teaching.*
