# Supplementary material for: Large-group virtual anatomy via a smart digital podium: a preliminary cross-sectional study of student perspectives toward remote anatomage teaching in a medical lecture hall
Source: BMC Med Educ. 2026 Jul 25;26:1684. doi: 10.1186/s12909-026-10019-x (PMC13685385; doi:10.1186/s12909-026-10019-x)
Supplement: Supplementary file 2 [file 12909_2026_10019_MOESM3_ESM.docx]

**Additional file 3 — STROBE Checklist (Cross-Sectional Studies)**

| **Section & topic** | **Item** | **Recommendation** | **Location / comment** |
| --- | --- | --- | --- |
| Title & Abstract | 1a | Indicate the study design with a commonly used term in the title or abstract | Title: "preliminary cross-sectional study." Abstract Methods: "A cross-sectional study, reported in accordance with the STROBE guideline..." |
|  | 1b | Provide an informative and balanced summary of what was done and what was found | Structured abstract with Background, Methods, Results, and Conclusions sections. |
| Background/Rationale | 2 | Explain the scientific background and rationale | Background, paragraphs 1–7: scalability gap, multimedia learning theory, COVID-era remote anatomy context, and study rationale. |
| Objectives | 3 | State specific objectives | Final Background paragraph: objectives stated explicitly. |
| Study design | 4 | Present key elements of study design early | Methods, Study design subsection: cross-sectional survey design stated. |
| Setting | 5 | Describe the setting, locations, and relevant dates | Methods: Kasturba Medical College, MAHE, Manipal, India; academic year 2024–2025; lecture date and 47-minute duration specified. |
| Participants | 6a | Give eligibility criteria and methods of selection | Methods, Participants: all 125 first-year MBBS students attending the lecture were eligible; questionnaire participation voluntary. |
| Variables | 7 | Clearly define all outcomes and exposures | Methods, Attitude questionnaire: all 13 items defined with response options and format. |
| Data sources | 8 | Give sources of data and methods of assessment | Microsoft Forms questionnaire; 13 items described in full. Cronbach’s α = 0.873 reported with explicit numeric coding scheme. Reflexive thematic analysis (Braun & Clarke) described. |
| Bias | 9 | Describe efforts to address bias | Anonymous questionnaire to reduce social desirability bias (acknowledged as only partially effective given proximity to faculty); novelty bias explicitly addressed in Discussion; non-response now stated as presumed rather than confirmed. |
| Study size | 10 | Explain how study size was arrived at | All students attending the lecture (n=125) invited; 94 responded (75.2%). |
| Quantitative variables | 11 | Explain how quantitative variables were handled | Ordinal responses reported as frequencies/percentages. Explicit numeric coding scheme reported for the 9-item alpha computation. |
| Statistical methods | 12a | Describe all statistical methods | Frequencies/percentages; Cronbach’s α with explicit coding and an interpretive caveat that alpha is descriptive, not confirmatory of unidimensionality. |
| Statistical methods | 12b | Describe subgroup analysis methods | Not applicable; no subgroup analysis performed. |
| Statistical methods | 12c | Explain how missing data were handled | No missing data for closed-ended items. For the open-ended item, 7/94 left it blank; handling described in Methods. |
| Participants | 13a | Report numbers at each stage | Attendance: 125; responses: 94 (75.2%); non-responders: 31 (reason presumed, not confirmed). |
| Participants | 13b | Give reasons for non-participation | Methods, Participants: voluntary non-completion presumed but not confirmed; alternative explanations (early departure, technical difficulty) acknowledged as unexcluded. |
| Descriptive data | 14a | Give characteristics of participants | Results, Participant characteristics: gender distribution reported. |
| Outcome data | 15 | Report numbers of outcome events/summary measures | All outcomes reported as n/N (%) throughout Results. |
| Main results | 16 | Give unadjusted estimates | Descriptive frequencies/percentages; no adjustment applicable. |
| Other analyses | 17 | Report other analyses done—e.g. analyses of subgroups and interactions, and sensitivity analyses | N/A — No subgroup, interaction, or sensitivity analyses were performed in this descriptive cross-sectional study. |
| Key results | 18 | Summarise key results referencing objectives | Discussion, Principal findings. |
| Limitations | 19 | Discuss limitations and bias sources | Limitations section: novelty bias, social desirability bias, absence of active comparator, non-response uncertainty, skill self-assessment validity, qualitative consensus/saturation limitations. |
| Interpretation | 20 | Give a cautious overall interpretation | Discussion frames results as preliminary; explicitly recommends a powered RCT with an active comparator arm. |
| Generalisability | 21 | Discuss generalisability | Limitations: single institution/batch/topic. Future directions: multi-institutional implementation recommended. |
| Funding | 22 | Give funding source and role of funders | Declarations, Funding: no external funding; APC covered under MAHE–Springer Nature agreement; no role in design/reporting. |
